# A novel uveitis model induced by lipopolysaccharide in zebrafish

**DOI:** 10.3389/fimmu.2022.1042849

**Published:** 2022-12-01

**Authors:** Xiao Xiao, Zhangluxi Liu, Guannan Su, Huan Liu, Wenhui Yin, Yuxuan Guan, Shixiang Jing, Liping Du, Fuzhen Li, Na Li, Peizeng Yang

**Affiliations:** ^1^ Henan International Joint Research Laboratory for Ocular Immunology and Retinal Injury Repair, Henan Province Eye Hospital, The First Affiliated Hospital of Zhengzhou University, Zhengzhou, China; ^2^ The First Affiliated Hospital of Chongqing Medical University, Chongqing Key Laboratory of Ophthalmology and Chongqing Eye Institute, Chongqing Branch (Municipality Division) of National Clinical Research Center for Ocular Diseases, Chongqing, China

**Keywords:** uveitis, zebrafish, inflammation, innate immunity, disease model

## Abstract

**Objective:**

Endotoxin-induced uveitis (EIU) is an important tool for human uveitis study. This study was designed to develop a novel EIU model in zebrafish.

**Methods:**

An EIU model in zebrafish was induced by intravitreal lipopolysaccharide (LPS) injection and was assessed dynamically. Optical coherence tomography (OCT) was used to assess infiltrating cells in the vitreous body. The histological changes wereevaluated using HE staining and immune cells were measured by immunofluorescence. The retinal RNA Sequencing (RNA-Seq) was used to explore the transcriptional changes during inflammation. RNA-Seq data were analyzed using time-course sequencing data analysis (TCseq), ClueGO plugin in Cytoscape, and Gene Set Enrichment Analysis (GSEA) software. Flow cytometry and retinal flat mounts were used to dynamically quantify the immune cells.

**Results:**

EIU was successfully induced in zebrafish following intravitreal LPS injection. Inflammation appeared at 4 hours post injection (hpi), reached its peak at 24 hpi, and then resolved at 72 hpi. Immunofluorescence confirmed that massive influx ofneutrophils into the iris and vitreous body, and activation of microglia as evidenced by ameboid-shaped appearance in the retina. Retinal RNA-seq during the EIU course identified four gene clusters with distinct expression characteristics related to Toll-likereceptor signaling pathway, cytokine-cytokine receptor interaction, NOD-like receptor signaling pathway, and extracellular matrix (ECM)-receptor interaction, respectively. Prednisone immersion inhibited the inflammatory response of EIU in zebrafish, whichwas confirmed by decreased neutrophils detected in flow cytometry and retinal flat mounts.

**Conclusions:**

We developed a novel EIU model in zebrafish, which may be particularly useful for gene-editing and high-throughput screening of new drugs for the prevention and treatment of uveitis.

## Introduction

Uveitis is one of the primary causes of visual impairment globally, accounting for 10-15% of all blindness cases in the world (1–3). Despite numerous studies conducted during the last decade, the underlying mechanisms of this disease have not yet been completely explained (4–6). Uveitis models have been successfully induced in rodents using uveitogenic antigens or LPS, which profoundly renewed our understanding about this disease (7, 8). EIU is a widely used model to simulate acute anterior uveitis (AAU), particularly human leukocyte antigen (HLA) B27 associated AAU (8–10). It has been used for the studies of pathological changes, immunological and genetic pathogenesis of uveitis (11, 12). However, the extensively used methods to study the molecular pathogenesis in mouse models, such as gene-editing, are somewhat expensive and time-consuming, and conventional methods for assessing inflammation are relatively single-dimensioned. Therefore, a superior uveitis model for multiple-dimensional study is urgently needed.

Zebrafish has become a favorable model for disease studies since it is a model organism with highly conserved genomes. In the context of immunity, zebrafish larvae develop innate immune cells within two days after fertilization, and adaptive immune system forms at approximately 3 weeks post fertilization (13, 14). Although the immune systems are relatively conserved across species, there are some differences between the mammals and zebrafish. Zebrafish is less susceptible to LPS, possibly due to the inability of the extracellular portions of zebrafish tlr4a and tlr4b to recognize LPS (15, 16). Still, it has the advantages of small body size, rapid life cycle, transparency, and simple breeding and genetic editing (17, 18). For example, in terms of gene-editing, the variant Ripk2^Asn104Asp^ in zebrafish augmented the innate immune response and NF-κB pathway in early-onset osteoarthritis by CRISPR technique (19). Pharmacologically, the morphology and locomotor behavior of zebrafish could facilitate the study on effectiveness and side-effects of medicines (20, 21).

Endotoxemia model in zebrafish has previously been described (22). However, a uveitis model in zebrafish has not been induced so far. Here, for the first time, we developed a model of EIU in zebrafish, which could be potentially applied for gene-editing and screening of novel drugs for uveitis.

## Materials and methods

### Animals

Zebrafish (Danio rerio) were bred and kept under conventional conditions (27.5°C, 14/10 hours of light/dark cycle, and brine shrimp twice daily feedings). The 6-month-old adult male zebrafish were used in this study. The following transgenic lines were used: (a) wild-type fish of the AB strain; (b) Tg(Mpx:GFP) zebrafish with Green fluorescent protein (GFP) expression by neutrophils; (c) Tg(coro1a:GFP;lyz:dsRed), in which microglia/macrophages express GFP only and neutrophils express both GFP and dsRed. We bought zebrafish lines from the China Zebrafish Resource Center (CZRC, China). Zebrafish experiments were approved by the Medical Ethics Committee of the First Affiliated Hospital of Zhengzhou University.

### Induction of EIU

Zebrafish were anesthetized by immersion in 0.1% buffered tricaine for 5 minutes. A microinjector (PLI-100A, WARNER INSTRUMENT, USA) was inserted at the iris limbal, and 0.2μL of 0.65% saline containing 0.2mg/ml, 0.5mg/ml or 1.0mg/ml LPS (O55:B5, L6529, Sigma Aldrich, USA) were injected into the left vitreous body of the zebrafish. The corresponding dosages calculated according to the animal’s body weight (0.5g/zebrafish) were 0.08μg/g, 0.2μg/g, and 0.4μg/g respectively. The opposite eye receiving an equal volume of 0.65% saline served as control. After resting for 20 seconds, zebrafish were returned into tanks with clean system water.

### *In vivo* imaging

Following anesthesia, OCT imaging was obtained by a Micron IV retinal imaging microscope (Phoenix Research Laboratories, Pleasanton, CA) at 0 hpi (Blank control), 4 hpi, 12 hpi, 24 hpi, and 72 hpi of LPS. The parameters were set according to the manufacturer’s protocol, and a full-length line scan was performed horizontally and vertically while the optic disc was focused.

### H&E staining

At 0 hpi, 4 hpi, 12 hpi, 24 hpi, 72 hpi, 5 days post injection (dpi) and 7dpi of LPS, zebrafish were anesthetized in 0.1% buffered tricaine, and whole eyes were enucleated using fine forceps. The eyes were then fixed overnight in 4% PFA (Sigma-Aldrich, USA). Slide preparation was carried out in the manner previously described (23). The slides were then stained by hematoxylin-eosin for histological analysis. Stained slides were viewed at low magnification (×20) using a Leica DM4 microscope (Leica GmbH, Germany). The numbers of infiltrating cells in the anterior chamber and vitreous cavity were collected from six slides, and quantified using ImageJ software.

### Immunofluorescence and quantification of inflammatory cells

At 0 hpi (Blank control), 4 hpi, 12 hpi, 24 hpi, and 72 hpi of LPS, zebrafish were anesthetized in 0.1% buffered tricaine. Cryosection was carried out in the manner previously described (23–25). Cryosections were blocked in 20% goat serum at 26°C for 30 min before being incubated with primary antibody (rabbit anti-zebrafish L-plastin (1:500, Genetex, USA), which is a leukocyte-specific form of the actin binding protein, and rabbit anti-zebrafish Mpx (1:200, Genetex, USA), which is specific for neutrophil). After washing in PBST for 30 min, the cryosections were incubated in secondary antibody Alexa-Fluor 488 (Jackson ImmunoResearch, USA) and Alexa-Fluor 594 (Jackson ImmunoResearch, USA) for 1 h, and washed in PBST for at least 30 min, then incubated in DAPI (1:1000, Sigma-Aldrich, USA), and finally covered with a cover ship. Stained slides were viewed at low magnification (×20) using a Zeiss LSM 980 confocal microscope (Carl Zeiss Meditec, Jena, Germany). The numbers of fluorescence positive cells were collected from 4 randomly selected fields, and quantified using ImageJ software.

### Visualization and analysis of microglia in the retina

Tg(coro1a:GFP;lyz:dsRed) zebrafish were used for visualization of microglia and neutrophils. At 0 hpi (Blank control), 4 hpi, 12 hpi and 24 hpi of LPS, zebrafish were anesthetized in 0.1% buffered tricaine. The eyes were removed and fixed for 1 hour in 4% PFA. After cornea, lens and sclera were removed, the retina was stripped from the choroid and cut four times into four equal quadrants from the edge toward the center. The retina around the optic disc (×20 or ×63 oil objective) was scanned using a Zeiss LSM 980 confocal microscope (Carl Zeiss Meditec, Jena, Germany) with z stacks. The GFP+/dsRed- cells indicate resting microglia in blank control, which show small somas with thin and ramified cells processes. After LPS injection, GFP+/dsRed- microglia morphology changed from ramified to ameboid-shape. Outlines of individual GFP+/dsRed- cells were manually traced and measurement of morphological feature was analyzed using the “NeuronJ” tool in imageJ software.

### RNA-Seq and data analysis

Retinal RNA was extracted from zebrafish at 0 hpi (Blank control), 4 hpi, 12 hpi, 24 hpi and 72 hpi of LPS, and at the aforementioned time points after saline injection. The enriched mRNA was fragmented into short fragments and reverse-transcribed into cDNA with random primers. The QiaQuick PCR extraction kit (Qiagen, Venlo, The Netherlands) was used to purify cDNA fragments, which were then end repaired, poly(A) added, and ligated to Illumina sequencing adapters. Sequencing was performed on Illumina HiSeq2500 by Gene Denovo Biotechnology Co. (Guangzhou, China).

Differentially expressed genes (DEGs, false discovery rate (FDR) ≤ 0.05 and |log2(fold-change)| ≥ 1) were determined using limma package (version 3.48.3). TCseq (v1.16.0) in the R package was used to conduct the time-course clustering analysis of DEGs. The functional enrichment analysis was performed by Cytoscape plugin ClueGO. To image the functional correlation between paths and genes, a kappa coefficient was calculated, based on gene overlap between paths or GO terms. The kappa threshold was 0.38 by default. The same color indicated functionally similar entries. The threshold for enrichment significance was P < 0.05. Global mRNA expression profiles were subject to GSEA using GSEA software (http://www.gsea-msigdb.org/gsea/index.jsp ). KEGG reference gene sets were downloaded from the KEGG database. The 1000 phenotype permutations were utilized in the enrichment analysis, and gene sets with nominal P < 0.05 and FDR < 0.25 were considered significant.

### Quantitative real-time PCR

Retinal RNA of zebrafish at 0 hpi (Blank control), 4 hpi, 24 hpi and 72 hpi of LPS were extracted using TRIzol (Invitrogen, Carlsbad, CA, USA). The cDNA was generated using EasyScript^®^ One-Step gDNA Removal and cDNA Synthesis SuperMix (Transgen, China). The QuantStudioTM3 Real-Time PCR Instrument (Thermo Fisher Scientific, USA) was used for PCR. Primers used are listed in **Supplementary Table S1**.

### Drug treatment

Zebrafish were immersed in 50μm prednisone (T8396, Topscience, China) immediately after LPS injection (prednisone-treated group), or immersed in 0.1% DMSO alone (vehicle-treated control).

### Sample preparation and flow cytometry

At 24 hpi of LPS, four retinas at each group were dissociated using 1 ml papain vial (Papain Dissociation System Kit, Worthington, USA) at 37°C for 30 min. The digestion was terminated using 3 ml washing buffer (5 ml fetal bovine serum (Gibco, USA) into 95 ml PBS). The single-cell suspension was filtered using a 40μm cell strainer (BD Falcon, USA). Flow cytometry was performed on FACS Celesta (BD Biosciences, USA). Acquired data were analyzed using Flow Jo version 10.1.

### Imaging of retinal flat mounts and quantification of fluorescent area

At 24 hpi of LPS, the Tg(Mpx:GFP) zebrafish were anesthetized in 0.1% buffered tricaine, followed by enucleating eyes. After cornea, lens and sclera were removed, the retina was stripped from the choroid and cut four times into four equal quadrants from the edge toward the center. The retina around the optic disc (×10) was scanned using a Zeiss LSM 980 confocal microscope with z series (Carl Zeiss Meditec, Jena, Germany). Percentages of the relative GFP-positive area were quantified using ImageJ software.

### Statistical analysis

GraphPad Prism 8 software (GraphPad Software Inc., San Diego, CA) was used to analyze the data. Normal distribution and equality of variance of groups were examined. For normal distributions, to analyze the difference between two groups, the independent t-test was used. To compare multiple groups, the One-way ANOVA was employed, followed by Dunnett’s multiple comparison test. If values did not pass the normality test, the Kruskal-Wallis test was used to compare among groups. Quantitative data are presented as mean ± SD, *P* < 0.05 was considered statistically significant.

## Results

### Histological and OCT imaging changes of EIU in zebrafish

**Figure 1:**
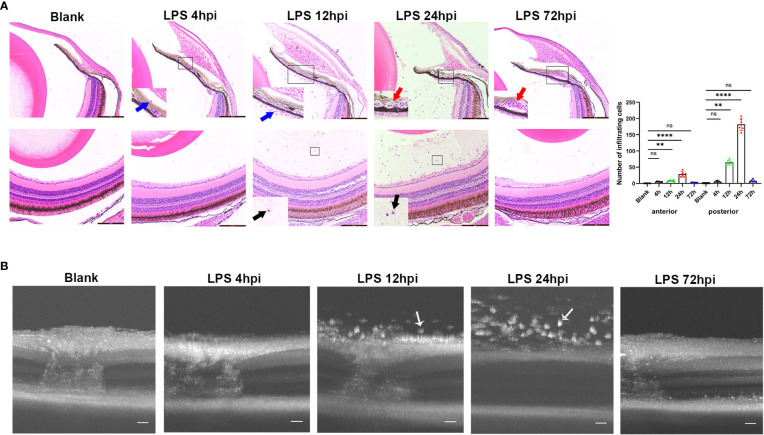
Inflammatory sign of EIU in zebrafish over time-course. **(A)** Left, H&E staining of eyes at different time points after LPS injection. Blue arrow, infiltrating cells around iris. Red arrow, vascular dilation. Black arrow, vitreous infiltration. Scale bars = 50μm. Right, quantification of inflammatory cells of anterior and posterior segments (mean ± SD; n = 6 eyes per group; **P < 0.01, ****P < 0.0001, ns, not significant; one-way ANOVA). **(B)** Serial OCT imaging of EIU at various time points after LPS injection. White arrow, highly reflex dots. Scale bars = 20μm. hpi, hours post injection.

Intravitreal LPS injection was performed at doses of 0.08, 0.2, and 0.4μg/g in zebrafish. There was no histological change in zebrafish receiving 0.08 or 0.2μg/g LPS, and those receiving intravitreal saline injection at 24 hpi (**Figure S1**). In 0.4μg/g LPS group, a mild inflammation as evidenced by sporadic infiltrating cells around iris was observed at 4 hpi. An obvious inflammation was then evidenced histologically by immune cells infiltrated the vitreous body and iris at 12 hpi, and this intraocular inflammation reached its peak at 24 hpi, showing massive influx of infiltrating cells and dilatation of iris vessels. At 72 hpi, the inflammation was alleviated and disappeared, but iris vascular dilatation still remained. The iris vascular dilatation gradually regressed at 5 and 7 dpi, leaving no signs of maladaptive structural remodeling (**Figure S2**). Overall, the highest number of cells infiltrating the anterior and posterior segments were seen at 24 hpi (**Figure 1A**). Therefore, the dosage of 0.4μg/g LPS was selected for further research. Consistent with histological observation, OCT imaging showed no obvious change in the eyes at 4 hpi of LPS compared to blank control. A large number of highly reflex dots in the vitreous body were observed at 12 hpi and followed by tremendous dots at 24 hpi. As expected, there was no highly reflex dot at 72 hpi (**Figure 1B**).

### Immune cell infiltration of EIU in zebrafish

**Figure 2:**
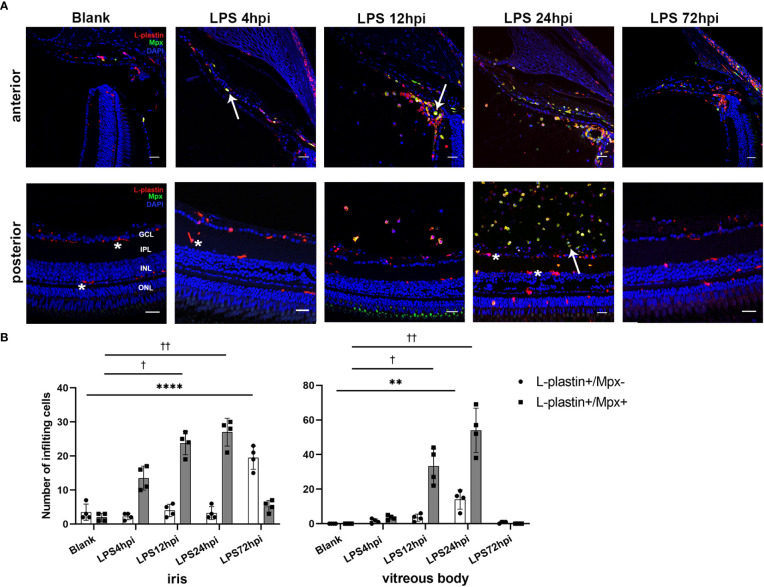
Distribution of immune cells during EIU inflammation process in zebrafish. **(A)** L-plastin (magenta), Mpx (green), and DAPI (blue) staining was seen in cryosections at 0 hpi (blank control), 4 hpi, 12 hpi, 24 hpi and 72 hpi of LPS. ONL, outer nuclear layer, INL, inner nuclear layer, IPL, inner plexiform layer, GCL, ganglion cell layer. Asterisk: L-plastin+/Mpx- cell. White arrow: L-plastin+/Mpx+ cell. Scale bar: 20μm. **(B)** Quantification of immune cells in the iris and vitreous body at each time point (mean ± SD; *P < 0.05, **P < 0.01, ****P < 0.0001, the number of L-plastin+/Mpx- cells were compared; ^†^ *P* < 0.05, ^††^ *P* < 0.01, number of L-plastin+/Mpx+ cells were compared, one-way ANOVA).

Immunofluorescent staining was performed to assess the influx of immune cells in the iris, vitreous body and retina during EIU process. As control, a small number of ramified L-plastin+/Mpx- cells, indicating retinal microglia, were observed in the retina of normal zebrafish. At 4 hpi, L-plastin+/Mpx+ cells appeared in the iris. The L-plastin+/Mpx- cells displayed shorter and thicker processes. More L-plastin+/Mpx+ cells infiltrated into the iris and vitreous body, accompanied by a small number of L-plastin+/Mpx- cells in and around the iris at 12 hpi. Subsequently, a tremendous influx of L-plastin+/Mpx+ cells infiltrated the vitreous body at 24 hpi, concomitanted with the L-plastin+/Mpx- cell morphology changed from ramified to ameboid-shape. At 72 hpi, the number of L-plastin+/Mpx+ cells almost declined to the normal level, while some L-plastin+/Mpx- cells still remained in the iris (**Figures 2A, B**). To further display the activation of microglia upon LPS challenging, we used Tg(coro1a:GFP;lyz:dsRed) zebrafish, in which neutrophils (GFP+/dsRed+) appear yellow and microglia/macrophages (GFP+/dsRed-) green. In line with the immunofluorescent staining, the GFP+/dsRed- ramified microglia only presented in the retina at 0 hpi of LPS (**Figure S3 A, E**). At 4 hpi of LPS, GFP+/dsRed- microglia showed enlarged cell bodies with shorter and thicker processes, accompanied by a small amount of GFP+/dsRed+ neutrophil exudation (**Figure S3 B, F, I**). At 12 and 24 hpi of LPS, GFP+/dsRed- microglia showed shorter and thicker processes and appeared ameboid in shape (**Figure S3 G, H, I**), accompanied with a massive infiltration of GFP+/dsRed+ neutrophils around the optic nerve (**Figure S3 C, D**).

### Transcriptomic hallmarks of EIU in zebrafish

**Figure 3:**
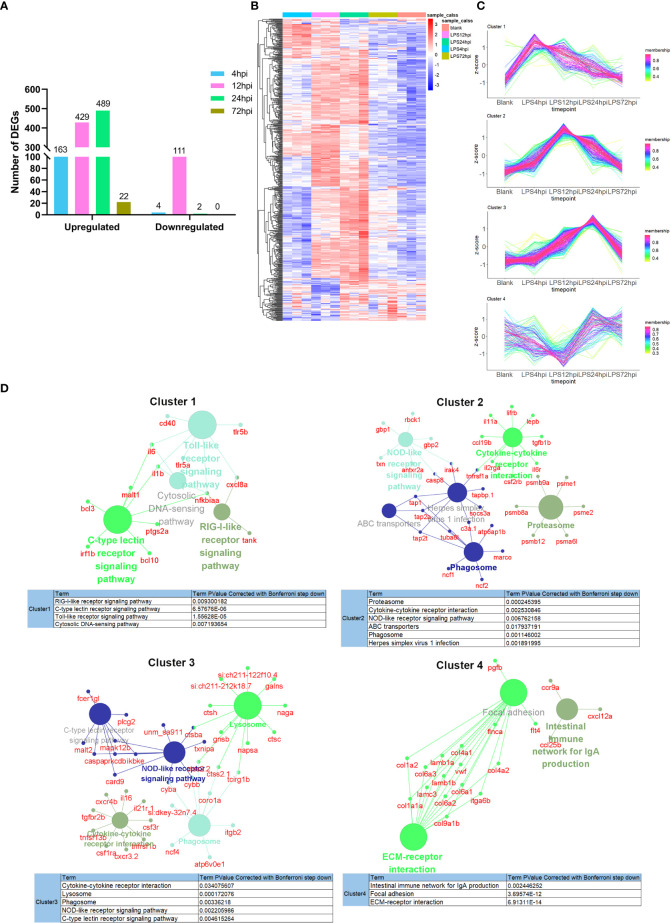
Transcriptomic traits of EIU in zebrafish. **(A)** Number of DEGs at 4 hpi, 12 hpi, 24 hpi and 72 hpi of LPS and saline. **(B)** Hierarchical clustering heatmap of DEGs at each time point. **(C)** The TCseq package was used to illustrate the patterns of dynamic changes in DEGs during EIU in zebrafish. **(D)** Functional enrichment analysis of DEGs in different clusters. Networks of pathways and genes in Cluster 1, Cluster 2, Cluster 3 and Cluster 4 were conducted by ClueGO and displayed by Cytoscape. Circles shown in the same color represent similar enrichment results.

Systematic RNA-seq experiment was performed to disclose the molecular mechanisms underlying EIU development in zebrafish. Heatmap for the Pearson’s correlation coefficient was performed to provide an overview for the relationships among samples. The Pearson’s correlations between the intragroup samples were all greater than 0.99, indicating that all intragroup samples were highly correlated (**Figure S4**). Differential expression level was set to fold change ≥ 2 (either upregulation or downregulation), and FDR should be less than 0.05. A total of 167, 540, 491 and 22 DEGs were observed respectively between LPS-injected and saline-injected groups at 4 hpi, 12 hpi, 24 hpi and 72 hpi (**Figure 3A**). Hierarchical clustering heatmap of DEGs showed gene expression returned to normal level at 72 hpi (**Figure 3B**). Time series cluster analysis by TCseq package showed four typical clusters (**Figure 3C**). Expression level of genes in cluster 1 peaked in 4 hpi, decreased steadily at 12 hpi and 24 hpi, and then declined to baseline at 72 hpi (**Figure 3C**, Cluster 1). Genes in this profile were strongly associated with Toll-like receptor signaling pathway, RIG-I like receptor signaling pathway, C-type lectin receptor signaling pathway and cytosolic DNA sensing pathway (**Figure 3D**, Cluster 1). Cluster 2 had a clear expression peak at 12 hpi, which then declined constantly at 24 hpi and 72 hpi (**Figure 3C**, Cluster 2). Genes in this profile were linked to NOD-like receptor signaling pathway, cytokine-cytokine receptor interaction, phagosome, proteasome, ABC transporters, and herpes simplex virus 1 infection (**Figure 3D**, Cluster 2). Expression level of genes in cluster 3 increased as the inflammation progressed, peaked at 24 hpi, and then decreased at 72 hpi (**Figure 3C**, Cluster 3). These genes were enriched in NOD-like receptor signaling pathway, lysosome, cytokine-cytokine receptor interaction, phagosome, C-type lectin receptor signaling pathway (**Figure 3D**, Cluster 3). Expression level of genes in cluster 4 decreased significantly at 12 hpi, but increased at 24 hpi, and restored to near baseline level at 72 hpi (**Figure 3C**, Cluster 4). Genes in this cluster were enriched in extracellular matrix (ECM)-receptor interaction, focal adhesion and intestinal immune network (**Figure 3D**, Cluster 4). We further performed GSEA to get insight into the biological roles of pathways involved in EIU development. The results showed that highly expressed genes were closely associated with innate immune response and NF-κB signaling pathway, whereas the low-expression genes were enriched in the ECM-receptor interaction and phototransduction (**Figure S5**).

### Quantitative RT-PCR validation

**Figure 4:**
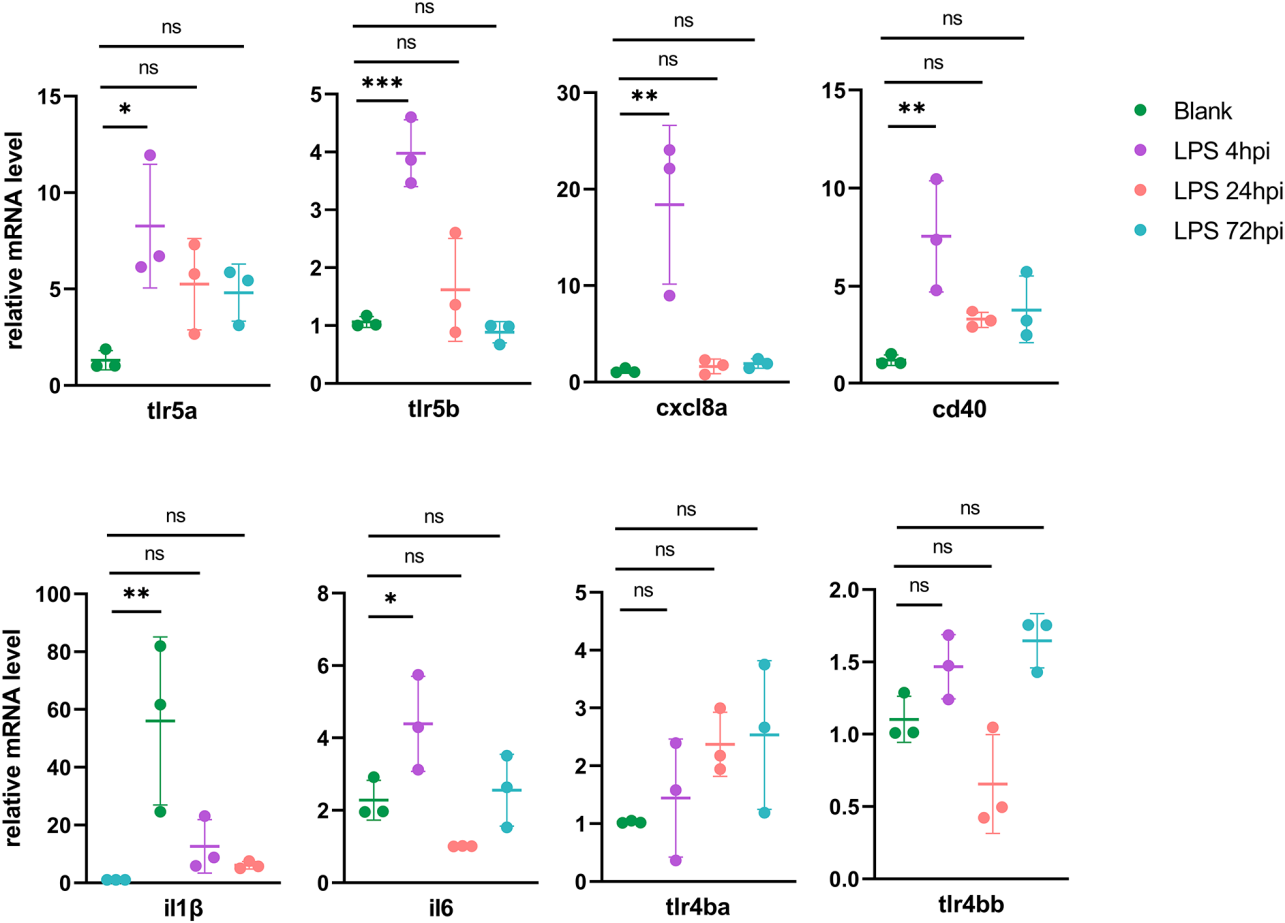
RT-PCR validation of DEGs in the initial of inflammation (mean ± SD; *P < 0.05, **P < 0.01, ***P < 0.001, ns, not significant; one-way ANOVA).

To validate the genes from pathways with most significant *P*-values in each cluster, 23 DEGs from RNA-seq were selected for RT-PCR validation, including Toll-like receptors signaling genes (*tlr5a, tlr5b, cxcl8a*, *cd40, il1β* and *il6*) in Cluster 1 (**Figure 4**), proteasome pathway genes (*psme1*, *psme2*, *psma6l*, *psmb8a* and *psmb9a*) in Cluster 2, lysosome pathway genes (*ctsh*, *ctsba*, *ctsc*, *napsa* and *galns*) in Cluster 3, and ECM-receptor interaction related genes (*itga6b*, *col1a1a*, *col1a2*, *col6a3* and *col9a1b*) in Cluster 4 (**Figure S6**). As Tlr4 is the receptor of LPS in mammals, we also tested zebrafish *tlr4ba* and *tlr4bb* expressions using RT-PCR, although they were not found in DEGs by RNA-seq. In the Cluster 1, all the six DEGs were upregulated at 4 hpi and then returned to normal level, while *tlr4ba* and *tlr4bb* expression showed no obvious change (**Figure 4**), consistent with the RNA-seq results (**Figure S7**). Besides, the expression of *psme1*, *psma6l*, *psmb8a* and *psmb9a* from Cluster 2, *ctsh*, *ctsba* and *napsa* from Cluster 3, and *itga6b*, *col1a1a* and *col1a2* from Cluster 4 showed similar profiles between the high-throughput RNA-seq and RT-PCR data **(Figure S6**).

### Effect of prednisone immersion on EIU in zebrafish

**Figure 5:**
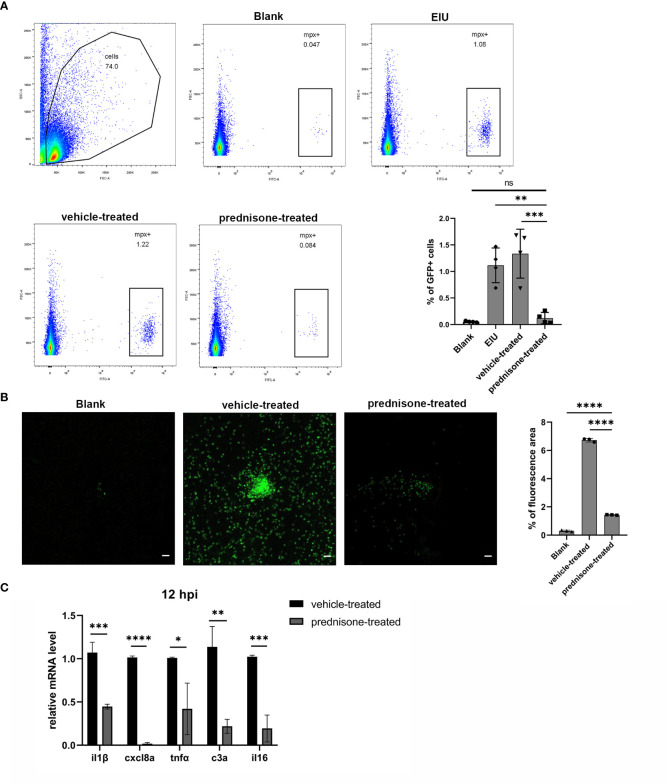
Prednisone immersion treatment inhibited EIU inflammation in zebrafish. **(A)** Live cells were separated out from the retina cell suspensions of Tg(Mpx:GFP) line. Left, Flow cytometry was used to analyze the GFP-positive neutrophils. Right, quantification of percentages of GFP-positive cells in different conditions (n = 4 per group; mean ± SD; ***P < 0.001; ****P < 0.0001; ns, not significant; one-way ANOVA). **(B)** Left, flat mounts of retina around the optic disc in Tg(Mpx:GFP) line. Scale bar, 20μm. Right, quantification of the relative GFP-positive area in retinal flat mounts in different conditions (mean ± SD; n = 3 retinas per group; ****P < 0.0001; one-way ANOVA). **(C)** Transcript levels of proinflammatory genes of retina in zebrafish treated with 0.1% DMSO (vehicle-treated group) or 50μm prednisone (mean ± SD; *P < 0.05, **P < 0.01, ***P < 0.001, ****P < 0.0001; unpaired t-test).

Prednisone immersion was used to evaluate its anti-inflammatory effect on EIU in Tg(Mpx:GFP) transgenic zebrafish line. Flow cytometry result of retinal cell suspensions showed that prednisone immersion significantly inhibited EIU inflammation as evidenced by substantially decreased GFP-positive neutrophils at 24 hpi as compared with vehicle-treated control group (0.1% DMSO immersion group) (**Figure 5A**). Furthermore, a similar result was observed by counting the relative GFP-positive area in retinal flat mounts, showing significantly decreased GFP-positive area in prednisone-treated group (**Figure 5B**). Retina from prednisone immersion group also showed decreased mRNA levels of proinflammatory genes including *il1β*, *cxcl8a*, *tnfa*, *c3a* and *il16*, indicating that prednisone was not killing neutrophils directly (**Figure 5C**).

## Discussion

In this study, we for the first time induced a zebrafish model of uveitis by intravitreal injection of LPS. In general, the intraocular inflammation of this model appeared at 4 hpi, exacerbated at 12 hpi, peaked at 24 hpi and resolved at 72 hpi, featured by distinct signaling pathways at different time points of the inflammation process. Furthermore, this intraocular inflammation was inhibited by prednisone immersion, suggesting that the EIU zebrafish model could act as a new approach to screen drugs for uveitis.

At the beginning of this study, we attempted different dosages of LPS to induce EIU in zebrafish at 0.08μg/g, 0.2μg/g and 0.4μg/g. There was no sign of intraocular inflammation according to histological analysis until the dose reached 0.4μg/g. It should be noted that this effective dose of LPS on zebrafish was 32-fold higher than that on mouse. The discrepancy of sensitivity to LPS between zebrafish and mouse might be related to the species difference between lower vertebrates and higher vertebrates (26). The studies have demonstrated that lower vertebrates, especially the fish, is tolerant to LPS stimulation due to the lack of the essential costimulatory molecular for LPS activation *via* Tlr4 (including myeloid differentiation protein 2 (MD-2) and CD14) (15, 16). Since eye is one of the first organs contacting the environment, the extracellular receptors might play an important role in resistant against bacterial infection. Despite the higher dose used in developing EIU in zebrafish, this new model has the advantage of low cost, smaller body size and rapid propagation to better satisfy experimental demands.

In the zebrafish EIU model, inflammatory cells began to infiltrated the iris at 4 hpi. A massive influx of cells was observed in the vitreous body and iris, accompanied by iris vascular dilatation at 24 hpi, and then spontaneously resolved at 72 hpi. The characteristic of rapid inflammation resolution in zebrafish was basically consistent with EIU model in mouse (8, 27). It would be of great significance to further explore the mechanism underlying the rapid resolution and short disease course, with the hope to provide new strategies for disease control and treatment in human. However, different from mouse, zebrafish has no ciliary body (28), thus the inflammatory cells of anterior chamber were observed around the iris. Besides its power to help investigate anterior uveitis, EIU is also a compelling tool to evaluate inflammatory responses at the posterior part of the eye. It is characterized by the breakdown of blood-retinal barrier (BRB) represented by an increment in adhesion of leukocytes to the retinal vasculature and infiltration of leukocytes into the retina/vitreous cavity (29–31). In the terms of immune cell type, myeloid cells including neutrophils and microglia/macrophages were the dominant populations of inflammatory cells in this zebrafish EIU model, consistent with mouse EIU model. Neutrophils were essential for evading infection *via* the generation of proteolytic enzymes and toxic intermediates (32). Besides, ramified L-plastin+/Mpx- microglia, the resident macrophage in the retina, morphologically transformed into ameboid-shaped during inflammation to response to inflammatory stimuli. These activated phagocytic microglial cells could facilitate the infiltration of leukocytes through the BRB and mediate adaptive inflammatory function in uveitis (33). However, it should be noted that L-plastin+/Mpx- staining is not specific to microglia, and macrophages migrated from other sites of the body might also exist.

At 4 hpi, genes related to Toll-like receptor signaling pathway mainly showed significant elevated expression. However, the Tlrs (*tlr5a and tlr5b*) expressed in zebrafish model were different from that in mammal EIU models. In mammals, *Tlr4* is the main receptor therein for LPS recognition with the participation of MD-2, LPS binding protein, and CD14 (34). Some recent studies have discovered the synergistic effect of *Tlr5* and *Tlr4* on triggering LPS-induced lung injury in mouse (35). Besides, *Tlr5* has also been proven to show potent anti-allergic effect on rodent (36). Although *tlr5* may be strongly associated with immunity or inflammation, the underlying mechanism of *tlr5*-mediated intraocular inflammation in the EIU model of zebrafish remains unclear and the exact roles of these upregulated *tlr5a* and *tlr5b* are worthy of further investigation.

At 12 hpi, upregulated genes were specifically enriched in phagosome and proteasome pathways. The phagosome is a vesicle formed by invaginating of the plasma membrane. Phagocytosis is a complex process, which could eliminate microorganisms and present them to CD4+ T cells and B cells. As previously reported, experimental autoimmune uveitis (EAU) could be alleviated by inhibiting the phagosome activation in macrophages, which prevent the activation of effector T cells and help maintaining the healthy ocular microenvironment (37). One important system closely related to the proteasome is the ubiquitin-proteasome system. Before a protein is degraded, it is first flagged for destruction by the ubiquitin conjugation system. The ubiquitin-proteasome is triggered by inflammatory stimuli and oxidative stress. In NF-κB pathway, it is necessary that the ubiquitinated IκB is degraded by proteasome to activate NF-κB. The deubiquitinase A20 has been reported to play a role in negative regulation of inflammation and immunity. In both Behcet’s disease (BD) and systemic lupus erythematosus, A20 expression was lower than that in health control, and it was especially downregulated in active BD patients (38, 39). These enriched-pathways in zebrafish were similar with those in mouse EIU model (40), and the therapeutic strategy based on signaling pathway could be further investigated in the future.

The downregulated genes at 12 hpi were mainly enriched in focal adhesion and ECM-receptor interaction. These pathways have also been reported to be downregulated in EIU model in mouse (40). The focal adhesion, regulator of the vascular intracellular tight junction proteins (TJs), maintained the BRB. TJs comprise many proteins, including claudins, occludins, and zonula occludens (41). The earliest molecular sign of BRB disruption is the aberrant expression of these marker proteins. BRB impairment leads to circulating leukocytes entering the retina and activating the innate immune response. In our study, substantial cells infiltrated the eye at 12 hpi meanwhile the expression of *claudin7* was decreased obviously.

Animal models are essential tools to help develop and validate new drugs. So far, proteasome inhibitor (42), immunosuppressant-loaded nanoparticles (43), and gut microbial metabolites (6) have been applied on murine uveitis models. In this study, we successfully validated the anti-inflammatory effect of prednisone on zebrafish EIU model, suggesting this model as an effective tool for future drug screening studies.

However, our study has some limitations. First, the specific contribution of tlr5 orthologs in zebrafish uveitis model is not yet clarified due to lack of available commercial antibodies against this molecule and its downstream signaling effectors in zebrafish, which awaits further investigation. Second, whether the anti-inflammatory effect of prednisone is through alternating tlr5 orthologs and whether smalls molecules targeting the TLR-signaling pathways could impose an effective protection against EIU also remain to be answered in the future. Third, considering RNA-seq results showed changes in NOD- and TLR-pathways, Crispr-Cas9 technique could be further introduced to study the involvement of these signaling pathways in the pathogenesis. Fourth, zebrafish is less representative to human in genome if compared with rodent models. Therefore results obtained based on zebrafish model should be interpreted more cautiously. Fifth, systematic inflammation is not examined in this study and is expected to be clarified in future studies. Sixth, since estrogen has been reported to influence cytokine production in mouse EIU model (44), we only used male fish in this study. The difference of EIU in zebrafish between male and female would be explored in the future.

In summary, we developed an EIU model in zebrafish which represents the characteristics of acute intraocular inflammation. We expect that this model will provide a complementary tool for the studies on pathogenesis and treatment of uveitis. The equations should be inserted in editable format from the equation editor.

## Data availability statement

The datasets presented in this study can be found in online repositories. The names of the repository/repositories and accession number(s) can be found below: Sequence Read Archive study accession code PRJNA891767.

## Ethics statement

The animal study was reviewed and approved by the Medical Ethics Committee of the First Affiliated Hospital of Zhengzhou University.

## Author contributions

PY, GS, and XX conceived and designed the experiments. XX, WY, and YG performed the experiments. XX, HL, and LD analyzed the data. SJ and NL contributed reagents. XX, ZL, FL, and PY wrote the paper. All authors contributed to the article and approved the submitted version.

## Funding

This work was supported by the Major Program of Medical Science and Technology Project of Health Commission of Henan Province (SBGJ202101011), the Natural Science Foundation Major International (Regional) Joint Research Project (81720108009), National Natural Science Foundation Key Program (82230032), and the National Natural Science Foundation Project (81970792), the Medical Science and Technology Project of Health Commission of Henan Province (SBGJ2020003031).

## Acknowledgments

The authors thank all participants in this study.

## Conflict of interest

The authors declare that the research was conducted in the absence of any commercial or financial relationships that could be construed as a potential conflict of interest.

## Publisher’s note

All claims expressed in this article are solely those of the authors and do not necessarily represent those of their affiliated organizations, or those of the publisher, the editors and the reviewers. Any product that may be evaluated in this article, or claim that may be made by its manufacturer, is not guaranteed or endorsed by the publisher.
