# Supplementary material for: A novel uveitis model induced by lipopolysaccharide in zebrafish
Source: Front Immunol. 2022 Dec 1;13:1042849. doi: 10.3389/fimmu.2022.1042849 (PMC9751191; doi:10.3389/fimmu.2022.1042849)
Supplement: Supplementary file 1 [file DataSheet_1.pdf]

Supplementary Material

1 Supplementary Figures and Tables

1.1 Supplementary Table

Table S1 Primer sequences used for RT-PCR in this study.

Gene	Forward primer sequence	Reverse primer sequence
cxcl8a	AAAGCCGACGCATTGGAAAA	TCCAGTTGTCATCAAGGTGG
il1β	GACCGGCAGCTCCATAAACA	CGAATCTTCATACGCGGTGC
tlr5a	CCGCGCACATCTGTGAAGTA	ATCCATCGTGTTGCCCTGTT
tlr5b	CATTAGTCGACGCGGTGTCA	CCTCACGCTTTCCATCCACA
tlr4ba	TTGACAACAACCCCACACGA	CTCACACACGTCACCCTTCA
tlr4bb	TGCTAGCTGCCTTCATTTCGT	ACTTATCCACGGTGAGGCCA
cd40	AGGCCAGATGACGATGTGAC	GCTCACAGGTGCGTTCTCAT
il6	ATGACGGCATTTGAAGGGGT	TCAGGACGCTGTAGATTCGC
ef1a	AAGCCTGGTATGGTTGTGACC	TCTCCAGCCACATTACCACG
psme1	GATTCGCAACACTTACGCCA	AGATAAGTCTCCCCTGGGCT
psme2	TGTCATCTAAACGTCTGCGTCT	ACAGTGACTGGCGGTAGTTTT
psma6l	GACAGTCGCTCTCAGGTTCA	CGGCACATCATAGCCGAACT
psmb8a	GACGCCTATTCTGGTGGTGT	ATCCCCTTCTTGTAGCGGTG
psmb9a	CCATCCGCTCAAAATGTCGG	ATGATGGTCGTCCCGGTTTT
ctsc	GCAACGGCGTACAGTTTTCA	ACTGTCCGCAGCAACAGTTA
ctsh	CTCGCTGTGGGTTATGCTGA	CAGTTGGTTCCCCAGGAGTT
ctsba	CGGCTGGACATAACTTCCGT	CAGGGAGTTTGGGACCCTTC
napsa	TGTTTGTCTGTCGGGCTTCA	GTAGTAGCGGCCGATGAACA
galns	ACCTCGCTGTCATGAACTGG	TGTGGCCAGTCACATTTCCA
itga6b	GCCCTGGTGGGTTATACTGG	AGAACGTTTGAAAAATCCACACT
col1a1a	CGGCCAGGTCTACAATGACA	ACTTCGTCGCACATTACGGT
col1a2	GGTAGCCCTGGCAGTTCTGG	TTTGGGACCAGGGAATCCAATGTTA
col6a3	ACCAGCTCCAGCTCCAGTTA	TGCATGGTAGTCCAGTGTCA
col9a1b	GGCTTTTCTGTCTTCGGGGA	AGGAGTCCCAGATCCAGTCC

1.2 Supplementary Figures

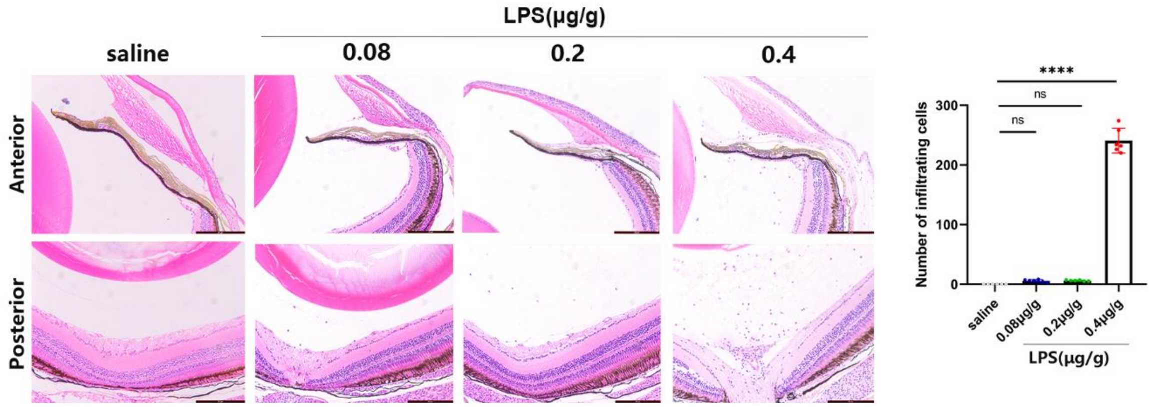

Figure S1. Ocular H&E staining in different dosages. Left, H&E staining of eyes receiving intravitreal saline and 0.08μg/g, 0.2μg/g, and 0.4μg/g LPS injection. Scale bar, 50μm. Right, quantification of infiltrating cells in eyes (n = 6 eyes per group; mean ± SD; ** *P* < 0.01, **** *P* < 0.0001, ns, not significant; one-way ANOVA). hpi, hours post injection.

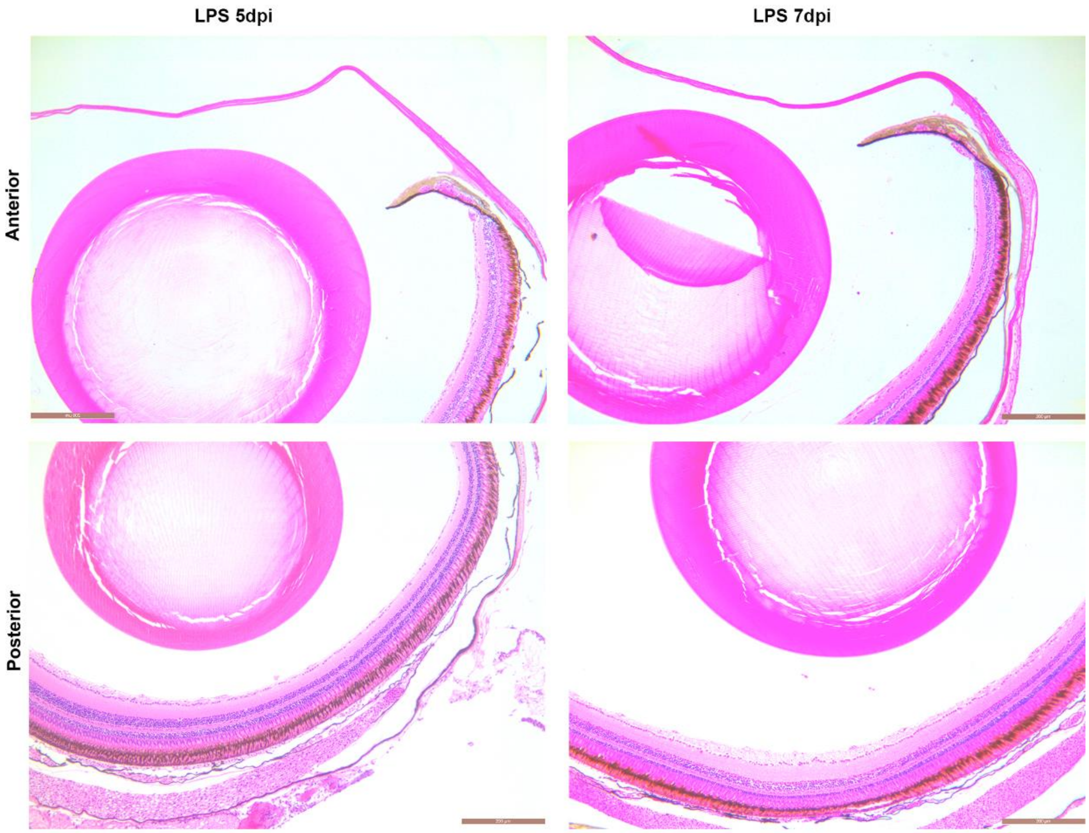

Figure S2. H&E staining of eyes at 5 days post injection (dpi) and 7 dpi of LPS. Scale bar, 100μm.

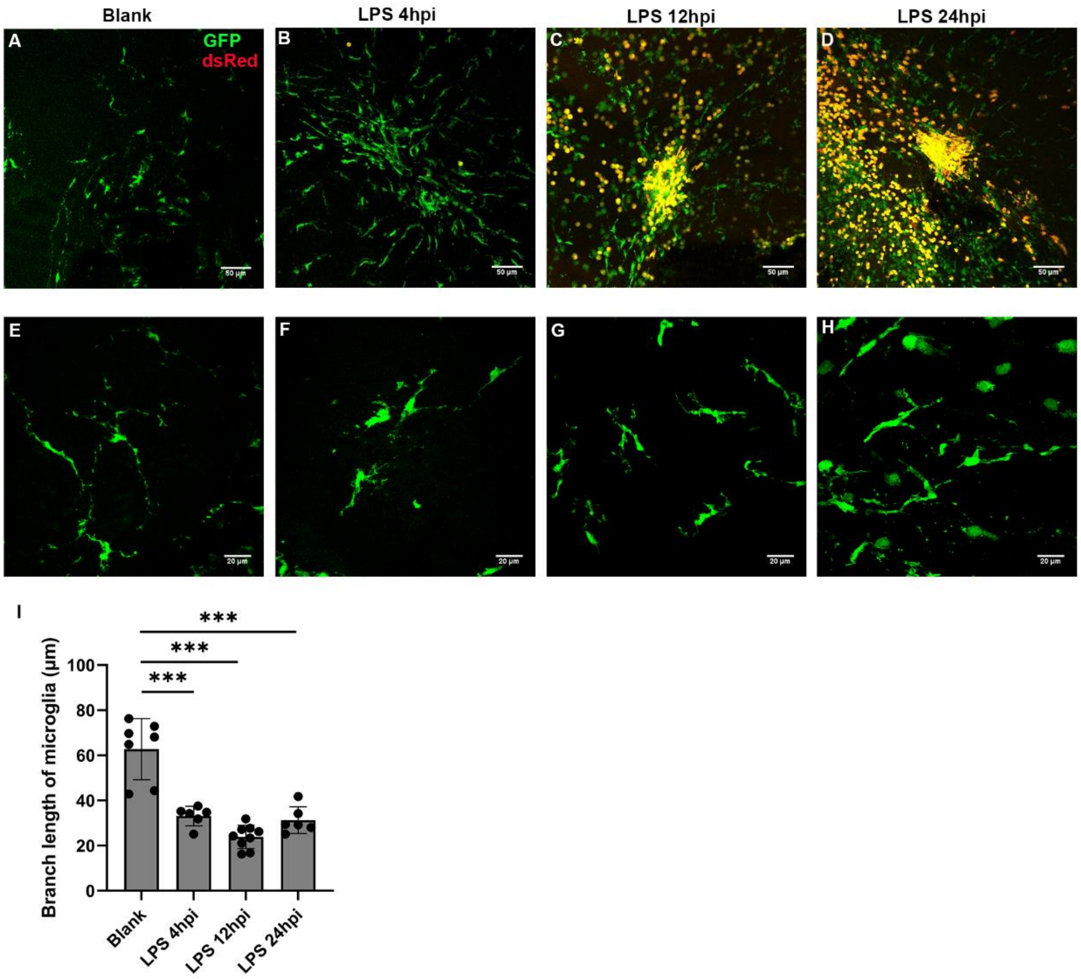

Figure S3. Intravitreal LPS administration enhanced the inflammatory reaction and activation of the microglia in the retina. (A-D) The retinal flat mounts of Tg(coro1a:GFP;lyz:dsRed) zebrafish at various time points after LPS administration. Scale bar, 50μm. (E-H) The (GFP+/dsRed-) microglia in a higher magnification. Scale bar, 20μm. (I) Quantification of branch length of microglia in the retina at various time points after LPS injection (mean ± SD; *** $P < 0.001$, one-way ANOVA).

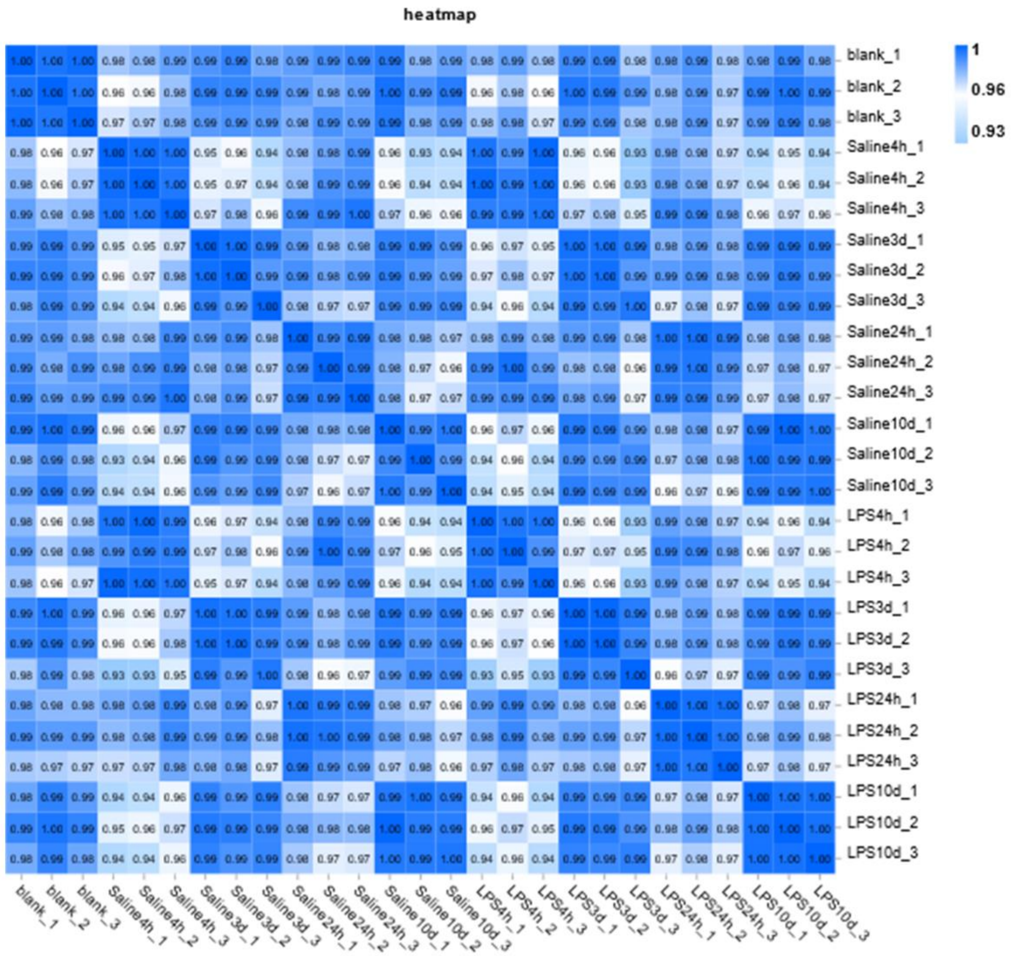

Figure S4. Heatmap of Pearson's correlation coefficient. A heatmap showed Pearson's correlation coefficient among different samples in retinal RNA-seq.

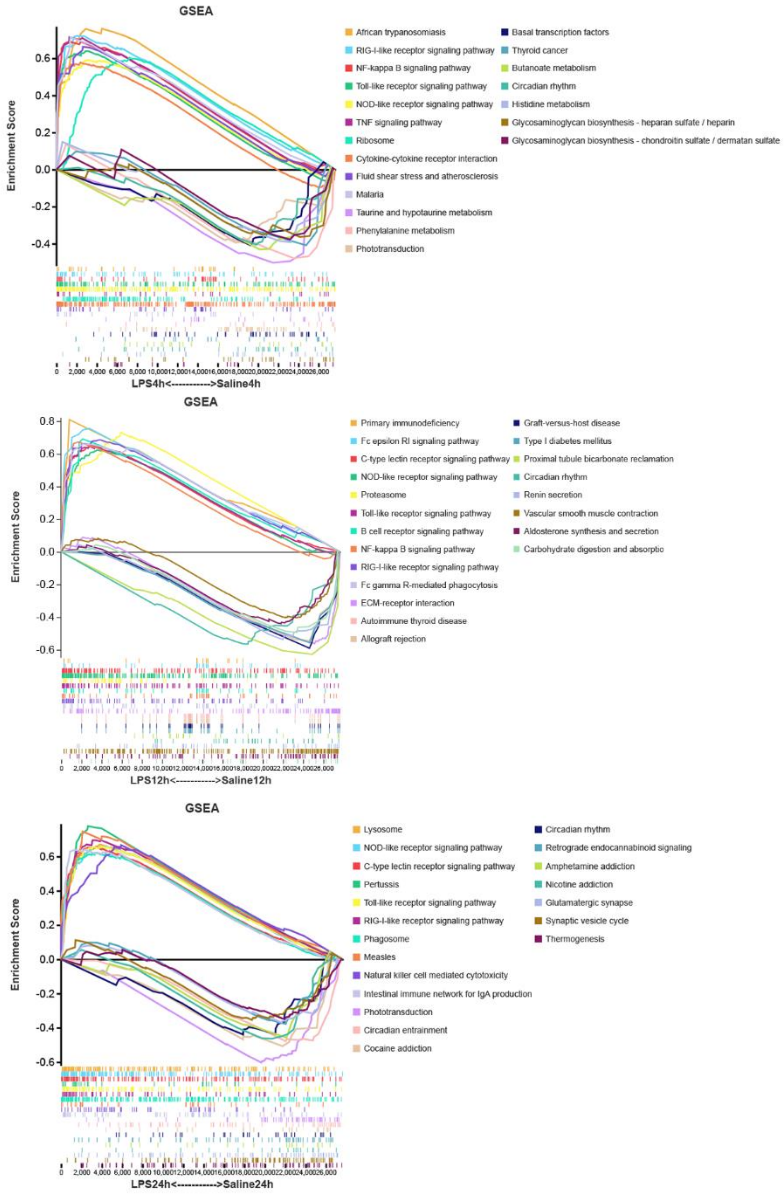

Figure S5. The GSEA of gene sets at different time points. GSEA-based KEGG-enrichment plots of gene sets at 4 hpi, 12 hpi and 24 hpi. hpi, hours post injection.

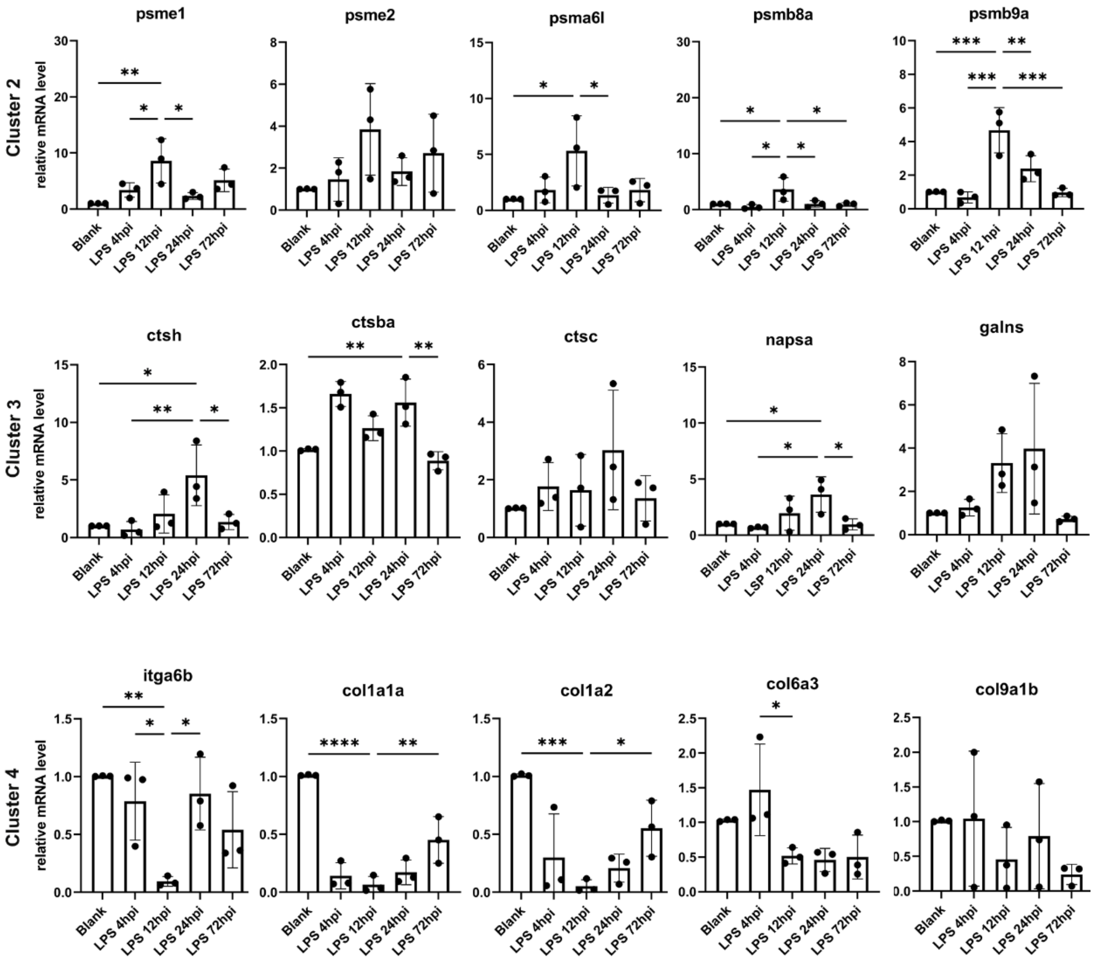

Figure S6. RT-PCR validation of DEGs in Cluster 2 (*psme1*, *psme2*, *psma6l*, *psmb8a* and *psmb9a*), Cluster 3 (*ctsh, ctsba, ctsc, napsa and galns*), and Cluster 4 (*itga6b, col1a1a, col1a2, col6a3 and col9a1b*) (mean ± SD; *$P < 0.05$, ** $P < 0.01$, *** $P < 0.001$, **** $P < 0.0001$; one-way ANOVA).

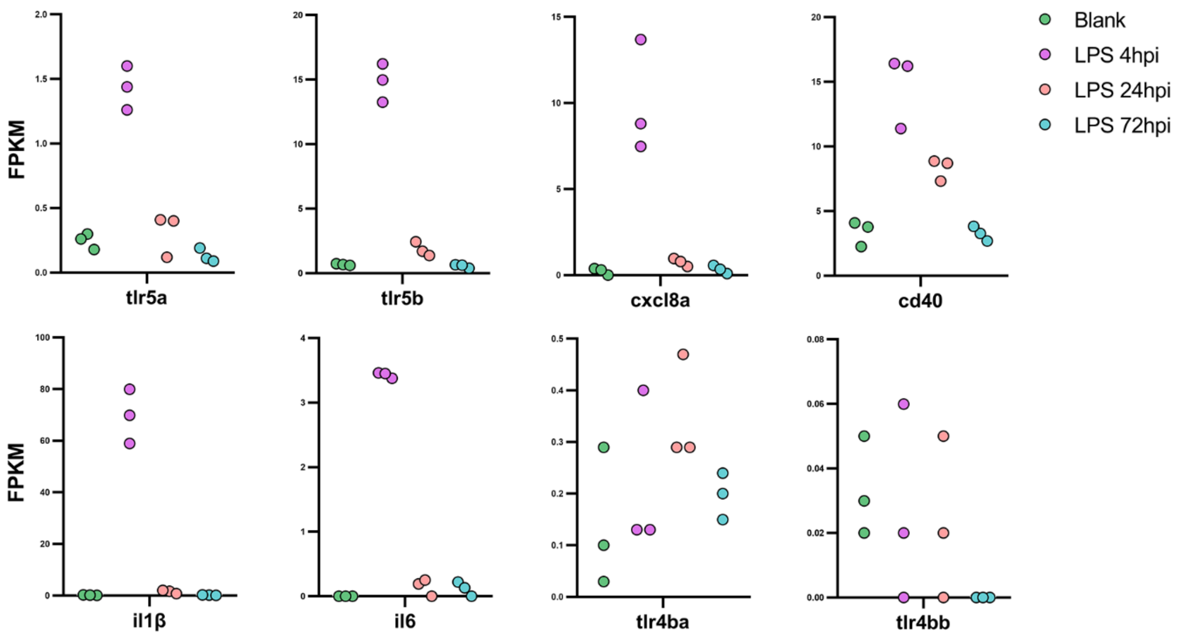

Figure S7. FPKM values in retinal transcriptome of EIU in zebrafish. FPKM values in retinal transcriptome for the 8 selected genes. All samples were analyzed in triplicate (n = 4 retinas per group). FPKM, Fragments Per Kilobase of exon model per Million mapped fragments.
